# A condition monitoring dataset based on electrical signals for a squirrel cage induction generator

**DOI:** 10.1016/j.dib.2025.112286

**Published:** 2025-11-13

**Authors:** Rafael Noboro Tominaga, Santiago Silveira Barbosa, Luan Andrade Sousa, Angelo dos Santos Lunardi, Rodolfo Varraschim Rocha, Sérgio Luciano Ávila, Bruno Souza Carmo, Renato Machado Monaro, Maurício Barbosa de Camargo Salles

**Affiliations:** a University of São Paulo (USP), São Paulo, Brazil; b Federal University of Mato Grosso (UFMT), Cuiabá, Brazil; c Federal Institute of Santa Catarina (IFSC), Florianópolis, Brazil

**Keywords:** Power generator, Electrical currents, Signature analysis, Behavior identification, Fault detection

## Abstract

The squirrel cage induction generator (SCIG) is still used in variable-speed wind turbines, although many other generator topologies are employed in renewable energy systems. Despite its mechanical robustness, low maintenance requirements, and the reduced complexity of control strategies typically employed in SCIG-based systems, the SCIG remains susceptible to internal faults, making early detection crucial for preventing severe damage and unexpected shutdowns. Monitoring critical components is essential. This dataset provides high-resolution electrical measurements from a SCIG under healthy and faulty conditions to support the development and validation of fault detection techniques.

The experimental setup consists of a laboratory test bench with an SCIG designed as a scaled-down version of a real wind turbine generator. Internal faults, including inter-turn and inter-winding short-circuits, were introduced in a controlled manner using a script that commanded a contactor to close the short-circuit for 400 ms. The faults used a single resistance of 2.6 Ω, and the number of affected turns was varied to represent different fault severities. The tests covered multiple steady-state operating points, with rotor speeds of 1200, 1500, and 1800 rpm and mechanical torques of 5.2, 6.4, and 8.0 Nm. Signals were sampled at 20 kHz and recorded during three-second intervals.

The dataset contains raw voltage, current, torque, and speed measurements from 24 distinct short-circuit scenarios plus one healthy condition, resulting in 225 .mat files. A Python interface supports visualization and analysis of the time-domain signals. The dataset can support signal processing studies aimed at enhancing short-circuit detection, serves as a resource for generator monitoring in wind turbine research, and assists in the development and testing of machine learning algorithms for time series classification. Although collected independently, this dataset complements another previously published by the same authors. The differences in machine topology and control approach justify the development of this new dataset.

Specifications Table

**Table:** 

Subject	Energy Engineering and Power Technology; Electrical and Electronic Engineering; Machine Design; Data Engineering; Signal Processing.
Specific subject area	Analysis of the electrical current signatures of a squirrel cage induction generator
Type of data	Table, Figure Raw data, not analysed, not filtered, and not processed. Processed data can be obtained by direct request to the authors
Data collection	Data acquisition was conducted in experimental benches equipped with industrial electro-electronic sensors positioned strategically to monitor the relevant variables. A data acquisition device was utilized to generate the files and reduce noise interference. A script was developed to automate the execution of the tests, however, at least two people were present at the location during the testing. Additionally, the files were systematically labeled based on the test conditions.
Data source location	Escola Politécnica, University of São Paulo, São Paulo, Brazil
Data accessibility	Repository name: SCIG-3phase-Dataset Data identification number: 10.5281/zenodo.17161986 Direct URL to data: https://github.com/InnovaPower/MitDev-Eletrica Instructions for accessing these data: documentation in the same github repository.
Related research article	None.

## 1 Value of the Data

• Signal processing researchers can use the data to develop methods that better highlight the effects of short-circuits through embedding and filtering techniques.
• Since the data come from an induction machine, they complement the synchronous machine dataset previously collected by the same authors, enabling various comparative studies for electrical machine researchers.
• Wind turbine specialists can include these data in studies focused on generator monitoring through current signature analysis.
• Because the data include labels, developers of machine learning algorithms for time series classification can easily use it in their research.
• Researchers interested in building datasets related to wind turbine failures, such as rotor imbalance, gear wear, or blade damage, can use this work as a reference to define appropriate methods for the phenomena under study.

## 2 Background

Although various other generator topologies are employed in renewable energy systems, the squirrel cage induction generator (SCIG) is still used in variable-speed wind turbines because of its mechanical robustness, operational simplicity, and low maintenance requirements [1]. Despite the simplicity of control strategies typically associated with SCIG-based systems, this generator type is still susceptible to internal faults, such as short-circuits, for which early detection is essential to prevent severe damage. The SCIG presents particular challenges for fault monitoring, primarily because its rotor is electrically inaccessible and does not involve any actively controlled variables [2]. Furthermore, instrumentation in practical SCIG installations is usually limited to measurements on the stator side, which constrains the observability of low-severity faults. Moreover, the effects of these faults on the measured signals are often mitigated or obscured by the control system itself, making their manifestation nearly imperceptible. As a result, data-driven diagnostic techniques require high-quality datasets that capture representative fault conditions. Nonetheless, publicly available experimental datasets comprising controlled fault scenarios in SCIGs remain limited. This work seeks to contribute to this research need by presenting a dataset obtained through the controlled insertion of inter-turn and inter-winding faults in a SCIG, aiming to support the development and validation of fault detection techniques.

## 3 Data Description

The data description presented here follows a similar approach to the one adopted for the permanent magnet synchronous generator (PMSG) dataset [3], with the differences highlighted in this section. Readers should consult this material whenever they have questions about any aspect not presented here. The tests, file labeling, and graphical interface are similar to what was presented in the PMSG dataset. It is not the purpose of this work to compare the datasets, as doing so could introduce interpretations and biases that would compromise the impartiality of this paper.

The dataset comprises 35 variables, with no missing values. These variables include both directly measured signals, acquired through physical sensors, and derived quantities, obtained through post-processing or estimation procedures. To facilitate interpretation, the variables are grouped according to their unit type. All signals were sampled at a rate of 20,000 samples per second. This high resolution is appropriate for capturing signal components typically found in electrical machines and power electronics systems, including harmonics and fault-induced distortions, which often occur in the kilohertz range. It also exceeds the Nyquist criterion by a wide margin, ensuring accurate signal reconstruction and preventing aliasing effects.

Table 1 presents a detailed list of these variables, including their corresponding descriptions and physical units. The table is organized into four columns: the first indicates the variable index; the second specifies the variable name; the third provides a brief description; and the fourth defines the unit of measurement.

**Table 1:** Description of the features.

Column	Feature	Description	Unit
1	t	Time elapsed after the start of the recording.	s
2	Ia	Current measured in phase A at the generator output terminals.	A
3	Ib	Current measured in phase B at the generator output terminals.	
4	Ic	Current measured in phase C at the generator output terminals.	
5	Id	Direct-axis current of the generator calculated according to current measurements.	
6	Iq	Quadrature-axis current of the generator calculated according to current measurements.	
7	Idref	Direct-axis current reference imposed.	
8	Iqref	Quadrature-axis current reference imposed according to speed measurements.	
9	Ifault	Fault current measured.	
10	Va	Voltage in phase A measured at the point between the generator and the converter.	V
11	Vb	Voltage in phase B measured at the point between the generator and the converter.	
12	Vc	Voltage in phase C measured at the point between the generator and the converter.	
13	Va_ref	Reference voltage calculated in phase A for converter output.	
14	Vb_ref	Reference voltage calculated in phase B for converter output.	
15	Vc_ref	Reference voltage calculated in phase C for converter output.	
16	Vd	Direct-axis voltage of the generator calculated according to voltage measurements.	
17	Vq	Quadrature-axis voltage of the generator calculated according to voltage measurements.	
18	Vd_conv	Direct-axis voltage calculated for converter output.	
19	Vq_conv	Quadrature-axis voltage calculated for converter output.	
20	Vdc	Voltage on the DC-link of the converter.	
21	Action_pi_d	Calculated PI controller action for direct-axis.	
22	Action_pi_q	Calculated PI controller action for quadrature-axis.	
23	Torq_ele	Calculated Electric torque.	Nm
24	Torq_mec	Measured Mechanical torque.	
25	Flux_est	Rotor magnetic flux estimated by the flux observer [4].	H
26	Flux_ref	Magnetic flux reference imposed for control.	
27	Theta_est	Electrical position of the rotor obtained through the flux observer [4].	rad
28	Theta_mea	Encoder position measured.	
29	Theta_kal	Encoder position estimated with a Kalman filter [5].	
30	Spd	Rotor speed measured.	rad/s
31	Fault_Relay	Fault relay command. A high level indicates that the command has been issued to close the contactor and start the short-circuit fault.	-
32	Da	Phase A converter duty cycle.	
33	Db	Phase B converter duty cycle.	
34	Dc	Phase C converter duty cycle.	
35	Package_loss	Package loss of CAN communication. This variable corresponds to a discrete integrator that increments continuously until successful communication occurs, at which point it is reset. Higher values indicate greater delays or interruptions in the CAN communication.	

**Fig. 1:**
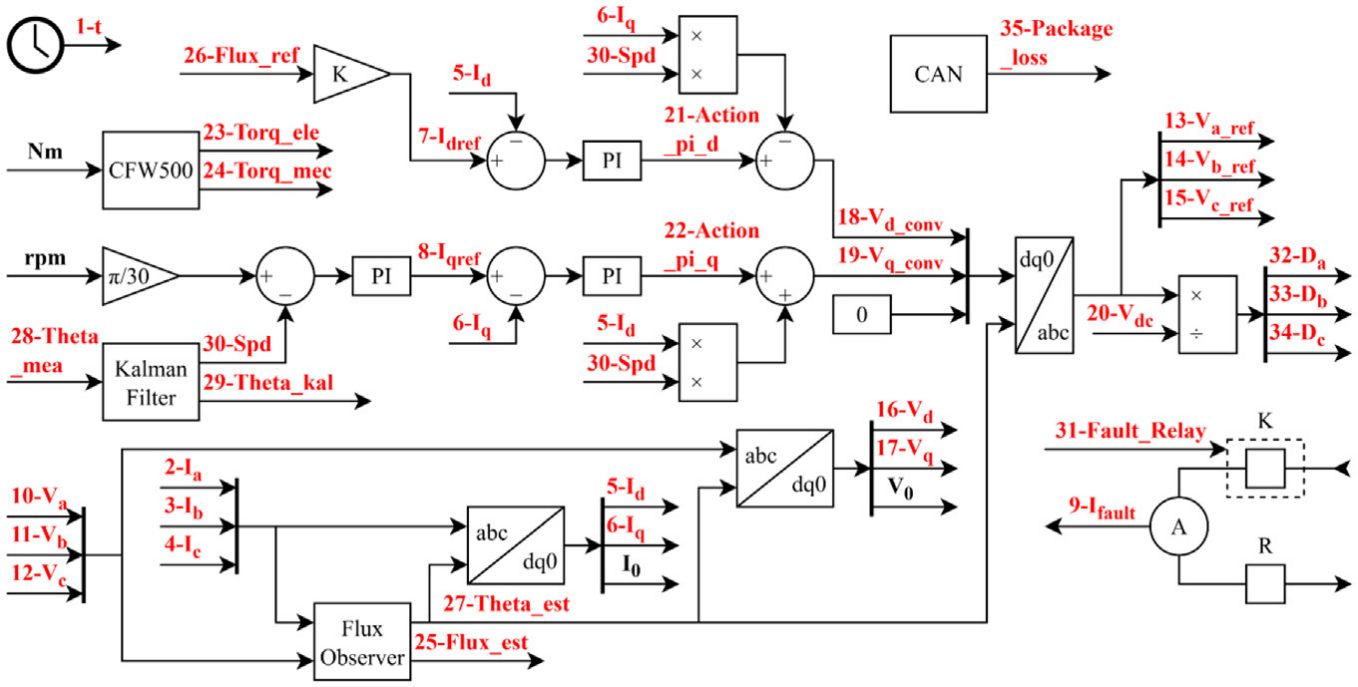
SCIG control and location of variables.

Fig. 1 complements this information by illustrating the location of each variable within the SCIG control diagram, serving as a visual reference for their function and position in the system. Unlike the PMSG, which allows direct measurement of the electrical angle using a rotor position sensor because of its synchronous operation and constant magnetic field, the SCIG requires a flux observer to estimate the rotor flux and determine the electrical angle indirectly. This fundamental difference increases the complexity of the control strategy, particularly for field-oriented control schemes.

**Fig. 2:**
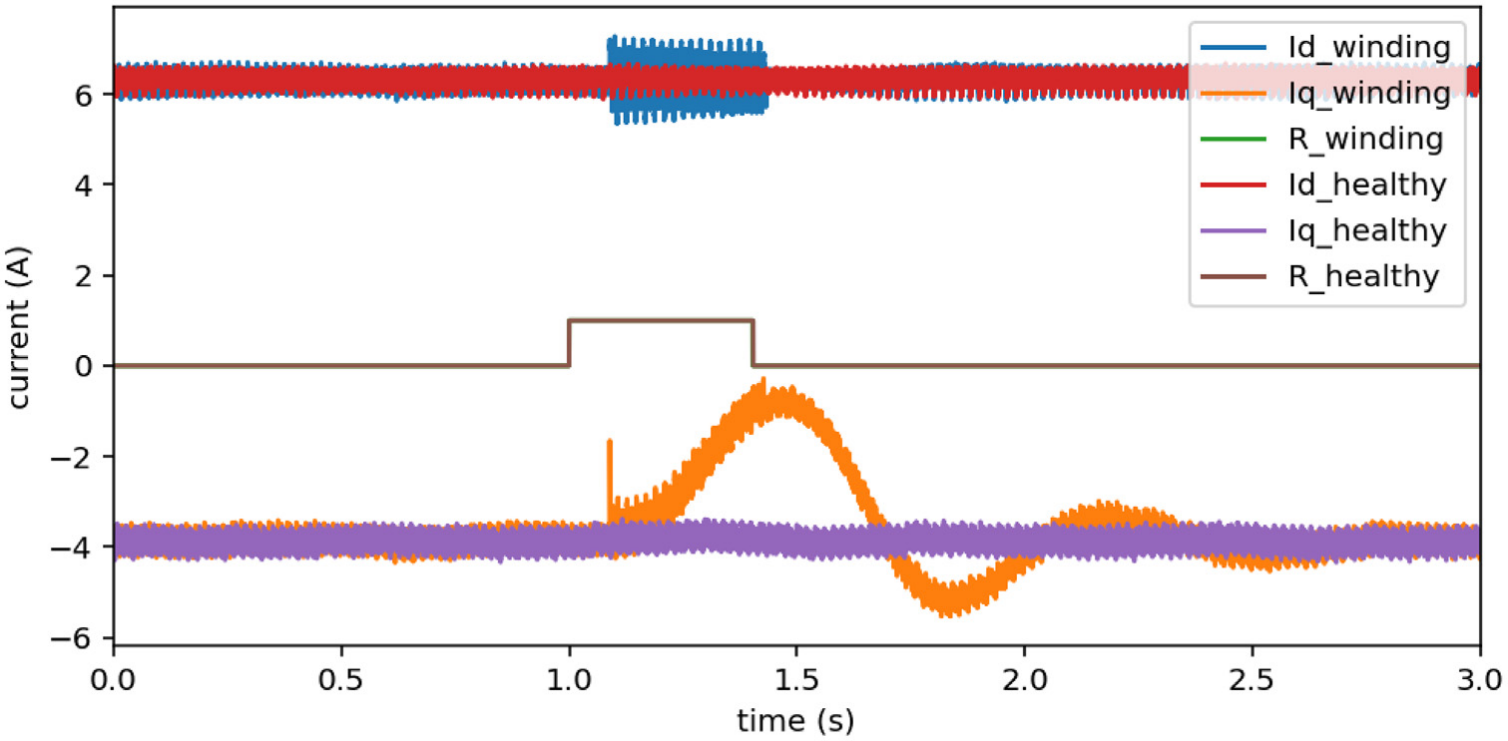
Comparative between D11 to D21 inter-winding short-circuit and healthy behavior using currents Id and Iq for 1500 rpm and 6.4 Nm.

Fig. 2 compares the direct and quadrature currents from two files in the dataset. The currents under healthy conditions remain stable throughout the 3-second window, showing no abrupt variations. In contrast, the currents in the file with an inter-winding short-circuit exhibit a significant disturbance immediately after the contactor closing command, which occurs at the first second. The visibility of this disturbance depends on the speed and torque of the generator, as well as on the severity of the short-circuit.

Each filename consists of a sequence of fields, separated by underscores, that encode specific test parameters. Table 2 presents a detailed description of each field in the filename.

**Table 2:** Filename composition description.

Field	Description	Possibilities
Sample type	Indicates a fault or normal (non-faulty) operating conditions.	FAULT, HEALTHY.
Fault type*	Specifies the fault type. In this dataset, two types are considered: TURNS (short-circuit between turns of the same winding) and WINDINGS (short-circuit between different windings).	TURNS, WINDINGS.
Derivations used*	Refers to the fault insertion points, corresponding to specific terminals along the windings.	D01_D04, D02_D03, and more 22 combinations.
Fault resistance*	Denotes the fault resistance in tenths of ohms.	R026.
Speed	Represents the rotational speed in revolutions per minute (rpm).	S1200, S1500, S1800.
Torque	Shows the mechanical torque in tenths of newton-meters.	T52, T64, T80.

⁎ Applicable only if sample type is FAULT.

## 4 Experimental Design, Materials and Methods

This section shows the changes in relation to the PMSG dataset [3]. Other devices not presented here, their connections, and the testing script are similar to those already described. It is important to emphasize that the test bench also inserts faults in the same way, using a script that controls a contactor to close the short-circuit for 400 milliseconds. The derivations available in the SCIG are identical to those in the PMSG and provide easy access to specific points of the windings, allowing the implementation of the short-circuits. The dataset includes the same list of short-circuits, covering 12 cases of inter-turn faults and 12 cases of inter-branch faults.

The system employs several sensors for monitoring and control purposes. A HBM T22 torque meter measures the mechanical torque transmitted from the motor to the generator, while an BRT50 encoder provides the angular position of the generator to the Imperix B-Box. Electrical signals are acquired through PEB8038 sensors. Additionally, a DIN 50A current transducer monitors current flow within the circuit.

**Fig. 3:**
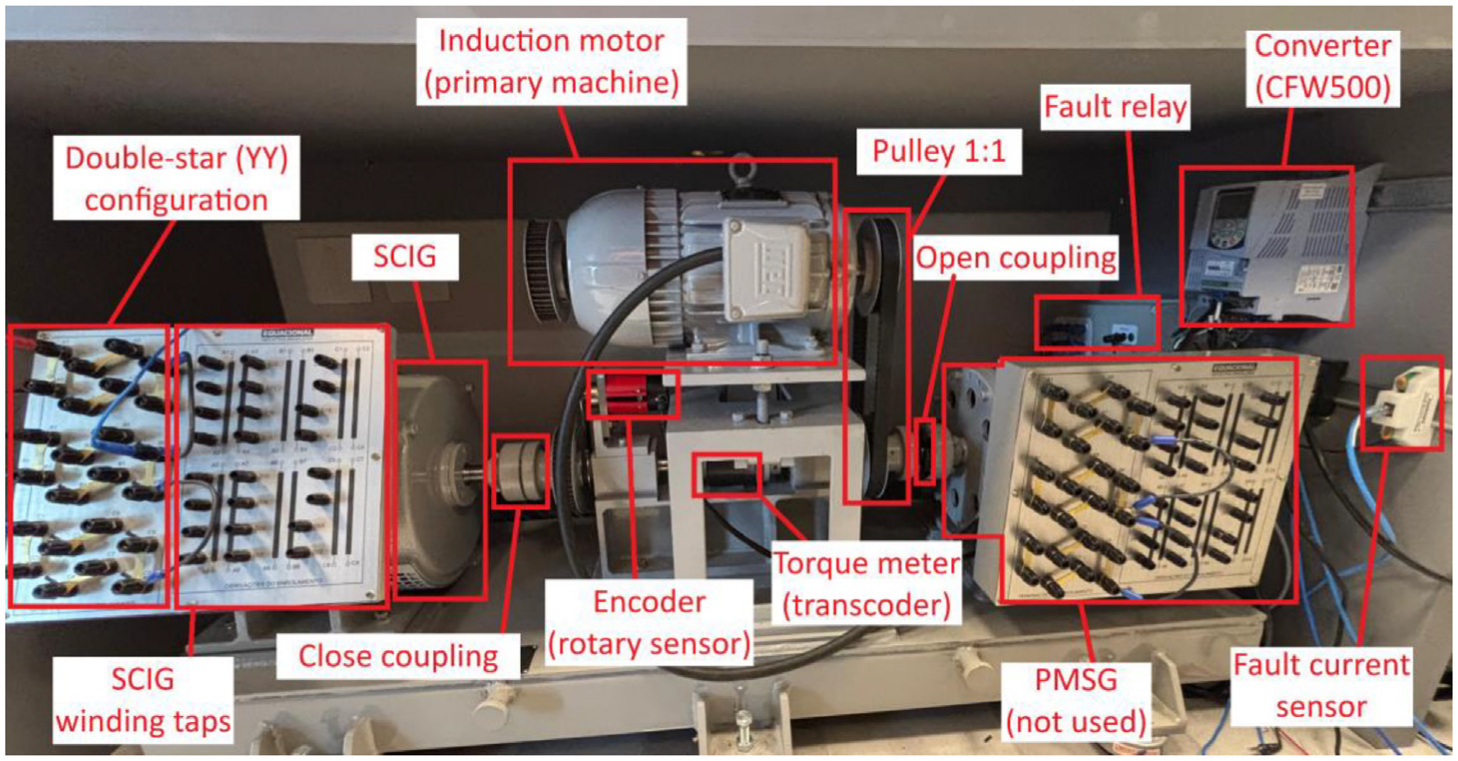
Setup for the SCIG system test bench.

The SCIG converts mechanical energy into electrical energy when driven above synchronous speed. In this condition, the rotor induces a current in opposition to the stator's rotating magnetic field, enabling the flow of electrical power from the generator to the grid. Table 3 presents the main design specifications of the SCIG and Fig. 3 shows the complete setup for the SCIG assembled at the test bench.

**Table 3:** SCIG specification.

Characteristic	Value
Manufacturer	Equacional [6]
Power	2.5 kW
Armature voltage	230 V - three-phase line voltage
Armature current	7.7 A (YY)
Nominal speed	1750 rpm at 60 Hz
Thermal insulation class	F - 155 °C
Regime of work	S1 (constant load)
Number of poles	4

**Fig. 4:**
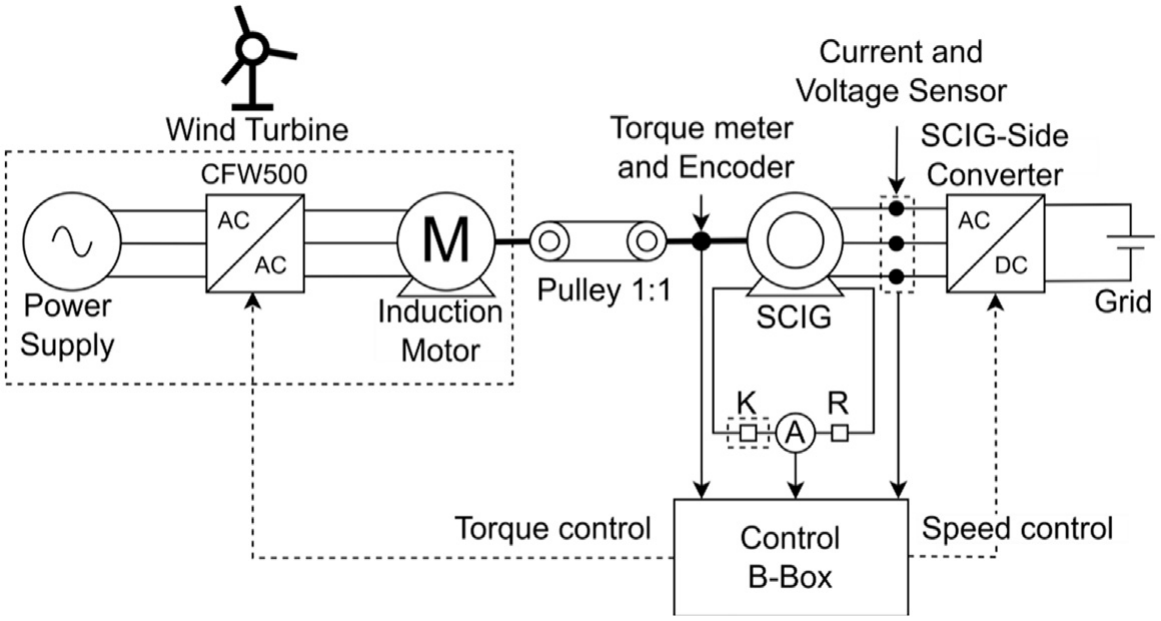
Block diagram of the SCIG system built on the test bench.

Fig. 4 shows the block diagram of the test bench, focusing on the SCIG setup. The system emulates a wind turbine through a power supply, converter, and induction motor that drives the SCIG. The SCIG connects to a converter, which in turn interfaces with the electrical grid. Two derivation points on the SCIG enable fault insertion through a contactor, an ammeter, and a fault resistance, all integrated into the Imperix B-Box for coordinated system control.

## Limitations

This dataset comes with limitations. Tests relied on a single low-power machine, since physical installation and safety constraints prevented experiments on high-power machines. Sensor readings include minor errors, which result from the inherent precision limits of the instrumentation. To avoid compromising the integrity of the equipment, the study controlled both the intensity and duration of the applied faults. The combinations of speed and torque covered only three values each, restricted by data storage capacity and time constraints. Inter-phase faults did not enter the study because of their severity and the high risk of permanent damage; their easier detectability also contributed to their exclusion. Finally, future applications described in the Value of the Data may require additional datasets to achieve satisfactory performance.

## Ethics Statement

Our research adheres to the ethical requirements for publication in Data in Brief, does not involve human or animal subjects, and no data has been collected from social media platforms.

## Credit Author Statement

**Rafael Noboro Tominaga:** Conceptualization, Methodology, Software, Validation, Formal analysis, Investigation, Data Curation, Writing - Original Draft, Visualization; **Santiago Silveira Barbosa:** Investigation, Writing - Original Draft, Visualization; **Luan Andrade Sousa:** Methodology, Software, Validation, Investigation, Resources; **Angelo dos Santos Lunardi:** Software, Validation, Investigation; **Rodolfo Varraschim Rocha:** Conceptualization, Methodology, Software, Validation, Investigation; **Sérgio Luciano Ávila:** Conceptualization, Writing - Review & Editing, Supervision; **Bruno Souza Carmo:** Conceptualization, Writing - Review & Editing, Resources, Project administration, Funding acquisition; **Renato Machado Monaro:** Conceptualization, Methodology, Software, Investigation, Resources; **Maurício Barbosa de Camargo Salles:** Conceptualization, Resources, Project administration, Funding acquisition.

## Declaration of Generative AI and AI-assisted Technologies in the Writing Process

During the preparation of this work, the authors used ChatGPT (OpenAI) in order to improve the clarity and grammar of the English language. After using this tool, the authors reviewed and edited the content as needed and takes full responsibility for the content of the published article.

## Data Availability

GithubSCIG-3phase-Dataset (Original data)
